# Step-by-step guide and checklists for selecting and conducting an evidence synthesis study using a framework for approaches and methods in evidence synthesis (FRAMES)

**DOI:** 10.7717/peerj.20897

**Published:** 2026-03-06

**Authors:** Alok Kumar Dwivedi

**Affiliations:** Center for Integrated Biostatistics and Epidemiology, Department of Biomedical Informatics, Biostatistics and Medical Epidemiology, University of Missouri School of Medicine, Columbia, MO, United States of America

**Keywords:** Scoping review, Systematic review, Meta-analysis, Checklists, Statistical analysis, Heterogeneity, Statistical methods, FRAMES

## Abstract

Findings from multiple studies are used to develop informed decisions for future research and clinical practices. Evidence synthesis methods, in general, are recommended for synthesizing findings from multiple studies. Among all evidence synthesis methods, a high-quality systematic review is required for answering a specific, focused research question from an abundance of literature. Although the choice of a systematic review among all other evidence syntheses depends on the formulated question related to estimation or hypothesis, and the availability of high-quality original studies, evidence syntheses not adhering to systematic review guidelines are also referred to as systematic reviews in the literature. Moreover, planning, designing, analyzing, and reporting a systematic review and meta-analysis requires multiple steps that are not included in the standard checklist documents, such as preferred reporting items for systematic review and meta-analysis (PRISMA) or meta-analysis of observational studies in epidemiology (MOOSE). It is also unclear to investigators how to properly select a type of evidence synthesis method and the steps involved in conducting a meta-analysis. Although multiple documents are available for selecting appropriate evidence synthesis methods, writing a protocol for a systematic review and meta-analysis, and conducting meta-analyses, there is no single report that consolidates all of these in a comprehensive framework. Given emerging challenges in the quality of published systematic reviews, this report aims to facilitate a framework for approaches and methods in evidence synthesis (FRAMES) to readers in (a) understanding types of evidence synthesis methods with specific focus to a systematic reviews and meta-analyses, (b) explaining the objectives of meta-analysis study, (c) describing the steps and resources in conducting a systematic review and meta-analysis, and (d) exemplifying the challenges and their potential solutions in a meta-analysis study. This report provides evidence-based biostatistics checklists for conducting a systematic review and meta-analysis, and writing steps for a systematic review study. I believe the adoption of this guidance document in research and training for the conduct and writing of an evidence synthesis study can have a far-reaching impact on producing high-quality evidence for research and clinical practices.

## Introduction

Clinical recommendations based on a single study may produce incorrect decisions (Pigott & Polanin, 2020). Therefore, multiple studies are conducted in different settings to validate the findings of original studies. An evidence synthesis study is often used to combine findings from multiple studies to develop informed decisions for clinical practices (Campbell et al., 2023). Some guidelines developed based on a single study were changed after conducting evidence synthesis studies (Bangalore et al., 2008; Neuman, Bosk & Fleisher, 2014). Some recommendations, such as low-dose computer tomography for lung cancer screening and the use of thalidomide with melphalan and prednisone for transplant-ineligible elderly multiple myeloma patients, are made using evidence synthesis methods while awaiting the results of large, randomized trials (Mandrekar & Mandrekar, 2011). Multiple excellent examples that change the practice guidelines in clinical medicine based on evidence synthesis can be obtained from Cochrane Collaboration reviews (Chandler & Hopewell, 2013; Guyatt, Mills & Elbourne, 2008; Moosapour et al., 2021). However, the number of studies published on the same topic is continuously growing with varying qualities, posing multiple challenges in conducting a high-quality evidence synthesis study. Multiple types of evidence synthesis approaches are available in the literature, and some of them are interchangeably used in the literature, creating confusion among researchers (Campbell et al., 2023; Moher, Stewart & Shekelle, 2015). The most common type of evidence synthesis is known as a systematic review. However, a well-planned, high-quality systematic review study requires time and resources that sometimes prohibit making a quick decision. In addition, multiple systematic reviews are being published on the same research question with varying qualities. Considering these challenges and values of an evidence synthesis, new methods of review synthesis, such as mapping review, scoping review, rapid review, living systematic review, and umbrella review, are emerging in the literature (Campbell et al., 2023). However, there is no clear guidance document available for selecting a specific type of evidence synthesis and the related steps in performing such studies in medical research.

A systematic review study among all evidence synthesis approaches is considered the top study design for producing the highest level of evidence. Systematic review is often required in medical research to generate clinical evidence for multiple reasons, including summarizing evidence from original research, increasing the external validity of research findings, increasing the statistical power and precision of research findings, and enhancing the reliability of the findings (Moosapour et al., 2021). However, the quality and credibility of evidence generated from systematic reviews are sometimes unreliable. A recent study highlights that approximately 80 systematic review studies are published per day (Hoffmann et al., 2021). Preferred reporting items for systematic review and meta-analysis (PRISMA) and meta-analysis of observational studies in epidemiology (MOOSE) are the recommended guidelines for reporting systematic review and meta-analysis studies (Brooke, Schwartz & Pawlik, 2021; Page et al., 2021). However, studies that do not adhere to the PRISMA and MOOSE guidelines are also referred to as systematic reviews. Multiple flaws have been identified in the conduct and reporting of systematic reviews (Nakagawa et al., 2017; Uttley et al., 2023). Prior guidance documents mostly focus on reporting and appraising systematic reviews, not selecting and conducting a systematic review and meta-analysis (Muka et al., 2020; Nakagawa et al., 2017; Shaheen et al., 2023). Moreover, planning, designing, analyzing, and reporting a systematic review and meta-analysis requires multiple steps that are not included in the available checklist documents, such as PRISMA or MOOSE. There are a few resources, such as the Cochrane Handbook and PROSPERO for protocol registration, that exist for selecting appropriate evidence synthesis methods, writing a protocol for a systematic review and meta-analysis, and conducting meta-analyses. However, they are scattered, and this report attempts to consolidate them in a framework. In addition, there is a gap in the literature for proper labeling of an evidence synthesis method, which helps in evaluating the quality of evidence generated from an evidence synthesis study, specification of the meta-analysis objective that helps in selecting approaches to meta-analysis methods, data collection elements and interpretations of findings, and evidence-based selection of statistical procedures that helps in evaluating the robustness of the findings obtained in a meta-analysis. Because of emerging challenges in the quality of published systematic reviews (Hoffmann et al., 2021), this report aims to facilitate a framework for approaches and methods in evidence synthesis (FRAMES) to readers in (a) understanding types of evidence synthesis with specific focus to a systematic reviews and meta-analysis, (b) explaining the objectives of a meta-analysis study, (c) describing the steps and resources in conducting a systematic review and meta-analysis, and (d) exemplifying the challenges and their potential solutions in a meta-analysis study.

### Types of review studies

There are many types of review studies, including narrative review, qualitative review, systematized review, overview, state-of-the-art review, critical review, literature review, mapping review, and other types of evidence synthesis available in the literature (Munn et al., 2018b). Among all, the most commonly conducted review study is known as a narrative review. A narrative review is useful for summarizing emerging trends, burdens, issues, challenges, mechanisms, and disease management for understanding the topic and identifying knowledge gaps. Typically, a narrative review is conducted on a topic with a broad scope without any statistical analysis, and the depth of reporting data and related discussions is under the control of the investigators (Baumeister & Leary, 1997; Greenhalgh, Thorne & Malterud, 2018). Sometimes, readers may be confused between a book chapter and a narrative review. A book chapter is the description of known evidence and interpretations. At the same time, a narrative review is the evaluation, assessment, interpretation, and summarization of gaps in existing research on a topic. Recently, a scale known as SANRA (Scale for the Assessment of Narrative Review Articles) was developed for quality assessment of narrative review articles (Baethge, Goldbeck-Wood & Mertens, 2019), and writing steps have been provided for narrative reviews (Green, Johnson & Adams, 2006). For a focused research question, the narrative review does not require a comprehensive search, and therefore, it does not generate any level of evidence. To generate the totality of evidence on a specific topic, a mapping review has been proposed. In a mapping review, it is required to conduct a comprehensive search related to each issue presented in the review article. Typically, a tabular presentation of all studies related to a specific problem is required. Although the mapping review is useful for generating evidence, mapping review involves a large number of articles to synthesize due to non-strict exclusion criteria and a lack of a precise research question (Campbell et al., 2023). A mapping review is useful for summarizing evidence when there is a broad topic with multiple objectives. Since the narrative and mapping reviews do not include a systematic approach to the selection of original studies and methods for evidence synthesis, the risk of bias in evidence selection and quality of evidence is compromised in these reviews. To address a specific objective and generate quality evidence through secondary data analysis, multiple evidence synthesis methods have been proposed.

### Types of evidence synthesis methods

**Figure 1:**
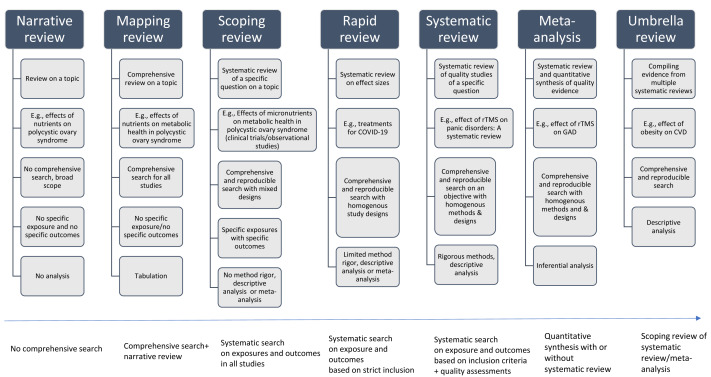
Definitions, examples, and methodological differences in evidence synthesis studies, including narrative and mapping reviews, along with scoping review, rapid review, systematic review, meta-analysis, and umbrella review.

**Figure 2:**
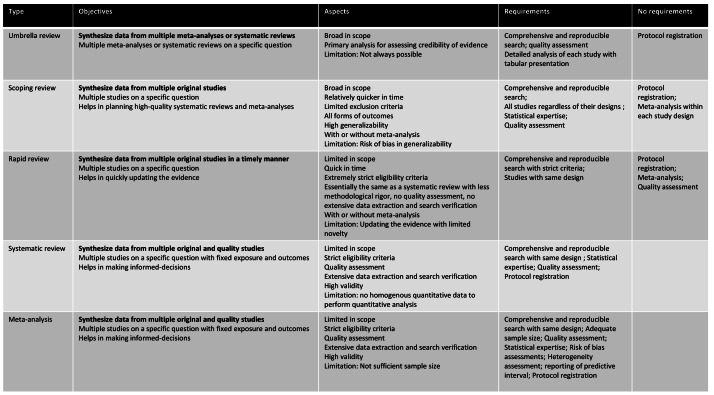
Strengths and limitations of different evidence synthesis studies.

An evidence synthesis study is a structured method for identifying multiple studies addressing a similar scientific question and synthesizing results from these studies to produce reliable evidence to make clinical decisions and policies. Evidence synthesis methods, in general, are recommended for synthesizing findings from multiple studies. The Cochrane Collaboration, an evidence synthesis organization, provides multiple resources, including handbooks and manuals for conducting a variety of reviews and evidence synthesis studies (https://www.cochrane.org/). The question can be related to effectiveness, experimental or intervention, estimation of prevalence/incidence, diagnostic test, etiology or risk factors, cost evaluation, psychometry/clinimetrics evaluation, predictive or prognostic evaluation, evidence from qualitative research or mixed methods, methodological evaluation, and policy evaluation (Kolaski, Logan & Ioannidis, 2023). Figure 1 displays the definitions, examples, and methodological differences in evidence synthesis studies, including narrative and mapping reviews, along with scoping review, rapid review, systematic review, meta-analysis, and umbrella review. The basic differences across evidence synthesis methods are methodological rigor and their reporting. Figure 2 provides the strengths and limitations of different types of evidence synthesis methods. The following sections provide detailed differences in different types of evidence synthesis methods.

### High-quality systematic review

A systematic review is required to answer a specific, focused, and well-formulated research question from abundant literature. The Cochrane Collaboration also facilitates methodological developments for systematic reviews (Chandler & Hopewell, 2013). In addition, several other guidance documents are available for conducting a systematic review and meta-analysis (Kolaski, Logan & Ioannidis, 2023; Moosapour et al., 2021; Zaccagnini & Li, 2023). However, some published systematic reviews are redundant, low-quality, unreliable, uninformative, contain methodological flaws, and do not fit the criteria of a systematic review or are mislabeled (Kolaski, Logan & Ioannidis, 2023). To differentiate the gold standard systematic review from other systematic reviews, we refer to it as a high-quality systematic review (HQSR). An HQSR requires an intensive, comprehensive, and reproducible search, comprehensive data collection, quality assessment of original studies, and descriptive statistical analysis of a specific and well-formulated scientific question based on strict eligibility criteria (Muka et al., 2020). The HQSR includes four aspects: (a) a comprehensive search to reflect the totality of evidence, (b) reproducible search to reflect the replicability of the systematic review study, (c) synthesis based on high-quality studies to reflect the robustness of the findings, and (d) synthesis based on homogeneous studies to reflect the high applicability of the findings. Due to these four steps, the conduct of an HQSR is resource-intensive, and it typically takes 1.5–42 months to complete the study, depending on the research question (Borah et al., 2017). An HQSR is mostly formulated for estimation or hypothesis-driven problems and related to intervention, prevalence/incidence, diagnostic test evaluation, etiology or risk factors, cost evaluation, and predictive or prognostic evaluation. The choice to use an HQSR among all other evidence synthesis methods might be based on a well-formulated question related to estimation or hypothesis, and, to some extent, on the availability of high-quality original studies. A systematic review can include evidence of varying quality without losing its methodological status, because its aim is to identify all relevant evidence. However, its conclusions should be based primarily on high-quality evidence. In contrast, evidence synthesis approaches that do not involve quality assessment, such as scoping reviews, do not draw conclusions based on evidence quality but instead describe the breadth and scope of the literature. Systematic reviews use evidence synthesis methods to summarize findings across studies. When statistical synthesis is performed (*i.e.,* meta-analysis), the primary aim is to include all available quantitative data, regardless of study quality. However, HQSR often dedicates a separate meta-analysis based on high-quality evidence, which is particularly important to their conclusion.

### Meta-analysis

A quantitative synthesis of homogeneous studies generated through HQSR is known as a meta-analysis. The number of adequate studies with consistent data is more deterministic in making decisions about conducting a meta-analysis compared to a systematic review. Researchers often name their study as a systematic review and meta-analysis without reporting descriptive analyses of all included original studies in the review. Since all data generated through an HQSR cannot proceed to a meta-analysis, studies that do not proceed to meta-analysis but meet the criteria for systematic review should also be analyzed to assess their influence on conclusions if authors claim their study is a systematic review and a meta-analysis (Muka et al., 2020). Therefore, if researchers claim a systematic review and meta-analysis as their study, then they may need to report results from descriptive as well as inferential data analyses. A meta-analysis is often completed within 10–24 months with intensive resources and technical expertise (Andersen et al., 2021).

### Rapid review

In some emerging areas or novel diseases, prompt evidence is required for making clinical decisions. Rapid review and living systematic review have been proposed to minimize the time and effort required in an HQSR or a meta-analysis. A rapid review is a type of evidence synthesis in which the methodological rigor of an HQSR, such as protocol registration, quality assessment, extensive data extraction, and search verification, is relaxed. A rapid review often requires 1–12 months for its completion (Tricco et al., 2015). Rapid review is useful for a research question where limited evidence or limited resources are available, or studies do not produce a sufficient sample size for conducting an HQSR and meta-analysis. A rapid review may include a meta-analysis approach to combine findings from recent, limited studies.

### Living systematic review

A living systematic review is a form of a rapid review on a specific topic, typically for a hypothesis-driven question that is continuously emerging owing to a novel disease or a novel area of research (Negrini et al., 2021).

### Scoping review

If investigators conduct a systematic search, they mostly refer to their study as a systematic review regardless of their quality. On multiple occasions, conclusions drawn from a systematic review or a meta-analysis are based on neither an ample number of high-quality studies nor on adequate homogeneous studies (Hoffmann et al., 2021). Moreover, findings from a systematic review or a meta-analysis that ignore the evidence from relatively low-quality studies or mixed designs may not be generalizable to a real setting. Considering the limited scope of an HQSR in the presence of heterogeneous studies with varying qualities, it is advisable to conduct a scoping review. Although scoping reviews are often interchangeably used as mapping reviews or systematic reviews, the purpose and aims of scoping reviews are different than mapping reviews or HQSR (Campbell et al., 2023). A scoping review without a precise research question is considered a mapping review. A scoping review is a form of evidence synthesis method in which studies with mixed designs, heterogeneous outcomes, or mixed qualities are included to provide an overview of a precise research question. If preliminary search yields (a) a good number of studies with mixed designs or heterogeneous assessments of outcome and exposure, or (b) a good number of studies but not sufficient quantitative or qualitative data for synthesis, or (c) any number of studies with mixed qualities, then the investigators may proceed to conduct a scoping review. In addition, the scoping review may be conducted for other purposes as described (Munn et al., 2018a). In a scoping review, a meta-analysis for a subset of studies may be conducted in addition to descriptive analyses (Tricco et al., 2016). A scoping review not only guides us to plan important future studies, including HQSR and meta-analysis related to the research question, but also produces a moderate level of evidence. Scoping reviews are also under the control of investigators concerning protocol registration, depth of data collection, and type of analysis, including descriptive analysis and meta-analysis (Pham et al., 2014). It is preferred to conduct a scoping review with meta-analyses rather than HQSR with a meta-analysis if investigators do not have adequate homogeneous studies or intensive resources.

### Umbrella review

Owing to multiple benefits of conducting an HQSR and meta-analysis, including no requirement of institutional review board approval, no recruitment of subjects, receiving more citations than any other study designs, and publications in relatively higher impact journals in some fields (Patsopoulos, Analatos & Ioannidis, 2005; Uthman et al., 2013), multiple systematic reviews and meta-analyses may be available on the same scientific question. In such a situation, an umbrella review may be performed to critically evaluate the quality of evidence generated in multiple systematic reviews and meta-analyses (Sadoyu et al., 2022).

### Selecting a specific review and evidence-synthesis approach

**Figure 3:**
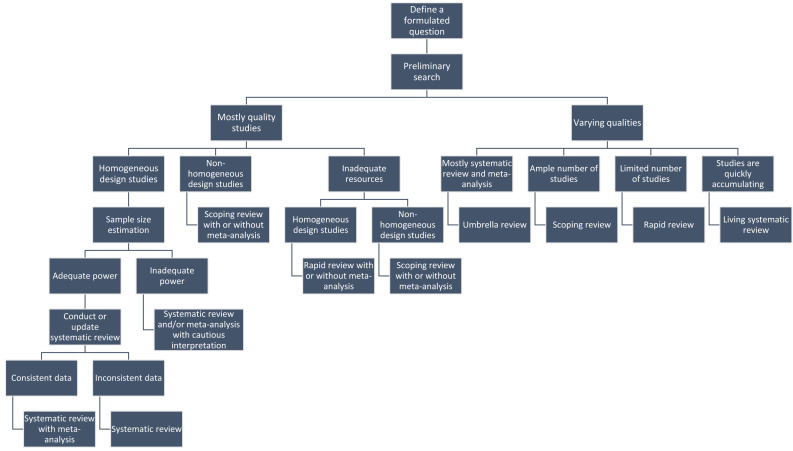
Approach for selecting an appropriate evidence synthesis study.

Figure 3 describes the approach for selecting an appropriate evidence synthesis study. Accordingly, the investigators first need to develop a research question. The research questions may include PICO (population, intervention, comparator, outcome), POSE (population, outcome, study design, exposure), PITS (population, index test, and target condition, study design), PCS (population, condition, study design), or other formats, depending on the type of research question (Kolaski, Logan & Ioannidis, 2023). If the specific question is related to a focused and precise objective, plan an evidence synthesis study; otherwise, conduct a narrative or other review study. Investigators can conduct a mapping review if the review study has more focused objectives. The plan of a specific evidence synthesis study depends on (a) study objective and hypothesis, (b) how quickly evidence is emerging and changing related to study objective, (c) time, resources, and expertise, (d) availability of original studies with same or different study design methods, and (e) number of studies with consistent and inconsistent data. After developing a precise research question, the investigators need to conduct a preliminary search related to the research question. If the search yields mostly good methodological quality studies in terms of general methodological rigors, then the investigators may prefer to conduct a systematic review with or without meta-analysis. Otherwise, they may plan to conduct a scoping or rapid review, depending on the homogeneity in study design among original studies. If most studies vary in their methodological qualities, then evidence synthesis methods other than systematic review and meta-analysis should be preferred. If the research question is related to an estimation or hypothesis, then the first preference should be conducting an HQSR and/or meta-analysis, provided an adequate number of high-grade original studies are available. If preliminary research yields consistent quantitative data from an adequate number of studies, then a meta-analysis should be performed; otherwise, an HQSR should be conducted. Suppose research requires the validity of findings proven from a meta-analysis, considering that new evidence or a literature search produces limited studies with consistent data. In that case, a rapid review may be preferred. An umbrella review may be performed for data from multiple systematic reviews or meta-analyses. If evidence continuously emerges on a specific question, then a living systematic review may be preferred over meta-analysis. If research questions are not related to an estimation or hypothesis, or preliminary search yields inconsistent data from mixed study designs or an insufficient number of studies, or if investigators do not have enough resources, then a scoping review should be conducted. The investigators should be cautious in labeling their studies as HQSR/meta-analysis if rigorous steps for synthesizing findings from multiple original studies are not performed. Instead, they may refer to their studies as a scoping review with or without meta-analysis, a rapid review, or simply an evidence synthesis study.

### Examples

Suppose investigators want to conduct an evidence synthesis on the effects of hormonal replacement therapy (HRT) on cardiovascular disease (CVD). For this topic, the investigators conducted a narrative review to describe the effects of different types of HRT (testosterone-based, estrogen-based, or estrogen with progesterone) on a variety of CVD outcomes and related underlying mechanisms (Dubey et al., 2004). Now it us up to the investigators to decide whether they want to discuss and summarize specific types of HRT in different age groups, related mechanisms, *etc.* in their narrative reviews (Hodis & Mack, 2022). Some investigators converted this narrative review to a mapping review by tabulating all evidence related to all types of hormonal therapies on CVD in postmenopausal women (Lowe, 2007). More specifically, some investigators also conducted an HQSR and meta-analysis to evaluate the effect of HRT on CVD in postmenopausal women using long-term randomized trials only or using RCTs and observational studies, or for specific CVD outcomes or mortality (Boardman et al., 2015; Yang et al., 2013). To produce evidence promptly, investigators also conducted a rapid review to evaluate the effects of HRT using recent long-term RCTs (Beral, Banks & Reeves, 2002). Considering heterogeneous studies on the role of testosterone therapy on different CVD risk markers, investigators conducted a scoping review on this topic (Britton & Beamish, 2023). Finally, investigators conducted an umbrella review in light of multiple systematic reviews on adverse effects associated with HRT or overall women’s health (Zhang et al., 2021).

### Types of meta-analysis

A meta-analysis can be conducted on participant data, known as individual participant data meta-analysis (IPD-MA) or aggregated participant data meta-analysis (AD-MA) from multiple original studies. Both IPD and AD meta-analyses produce consistent estimates and conclusions for moderate-sized studies. However, IPD-MA should be preferred over AD-MA when feasible, as it helps in performing detailed data analysis, including safety outcome, subgroup, interaction, and adherence analysis. Although the data-sharing mechanism allows us to conduct more IPD-MA, it is a resource-intensive study relative to AD-MA, as data acquisition on individual participants is sometimes tedious, and missing negative studies may adversely impact the outcome (Lyman & Kuderer, 2005; Tudur Smith et al., 2016).

### Why conduct an AD-MA?

A meta-analysis of high-quality studies, especially randomized trials, ranks top for producing the highest level of evidence in the pyramid of study designs or review studies (Haidich, 2010). Moreover, a high-quality meta-analysis may also lead to high-impact publications (Uthman et al., 2013). Since published HQSR and meta-analyses often receive higher citations compared to other types of studies, including reviews (Patsopoulos, Analatos & Ioannidis, 2005), the investigators are sometimes tempted to publish systematic reviews and meta-analyses without paying attention to the quality of their studies. However, the investigators should not plan any medical studies based on citation counts. Moreover, the meta-analytic findings helped in changing the clinical guidelines in some situations. Despite these benefits, investigators sometimes poorly execute AD-MA due to a lack of proper understanding of the type, objectives, requirements, resources, steps, and related challenges involved in meta-analysis studies (Uttley et al., 2023). In the following subsections, I highlight the steps, challenges, and potential solutions for conducting an AD-MA.

### Is meta-analysis an original investigation?

Sometimes, the investigators’ enthusiasm diminishes, considering meta-analysis as a review study, not an original investigation. However, most of the properties required for an original investigation are satisfied with a meta-analysis. One of the main criteria for an original investigation is to generate some new data or findings through a structured protocol. A meta-analysis is a systematic approach that requires following a structured protocol delineated in the PRISMA and MOOSE guidelines (Brooke, Schwartz & Pawlik, 2021; Page et al., 2021). In addition, a meta-analysis generates new data and findings that cannot be effectively and efficiently assessed with other approaches. For example, if investigators want to summarize the effectiveness of various interventions or doses on a disease outcome, then it would not be feasible with a randomized trial design. However, a meta-analysis can be utilized to determine the efficacy of interventions through network analyses or optimum doses through dose–response analyses. Other properties of an original investigation, such as data abstraction, statistical approaches according to study objectives, and data-based conclusions, are also required in a meta-analysis. In addition, the collection and presentation of data on the critical evaluation of each article is also required in a meta-analysis. Contrary to other studies, where it is sometimes hard to evaluate the authorship contributions, it is much easier to identify authorship based on contributions toward screening of articles and data collection, quality assessment, data analysis, and results presentation (Krnic Martinic et al., 2019). A relatively large proportion of journals, including several top journals such as Radiology, JAMA, and BMJ, consider a meta-analysis as an original investigation. In studies on a survey of editors, the majority recognize a systematic review and meta-analysis as an original article (Krnic Martinic et al., 2019; Meerpohl et al., 2012).

### Objectives of a meta-analysis

According to the clinical purpose of meta-analysis, different types of meta-analysis, including meta-analysis for resolving contradictions, meta-analysis for establishing effect size and increasing generalization of the findings, or updating the evidence, network meta-analysis, cumulative meta-analysis, dose-response meta-analysis, and meta-analysis for identifying risk factors or modifiers, have been conducted in medical research. The specification of an objective of a meta-analysis study guides us to decide appropriate statistical methods, including primary methods of analysis with fixed effect or random effects models for effect size calculation and regression analyses, and approaches of meta-analysis, including cumulative, network, or dose–response analyses, and the type of data collection elements in the study. The most common objective of a meta-analysis is to resolve conflicting findings obtained across studies (Wang et al., 2021). We conducted a meta-analysis to resolve conflicting associations between maternal polycystic ovary syndrome (PCOS) and adverse neuropsychiatric outcomes in their children (Dubey et al., 2021b). A meta-analysis was conducted to establish the effect size of repeated transcranial magnetic stimulation intervention on generalized anxiety disorder (Cox et al., 2022). In clinical medicine, different treatments or interventions are used to establish the efficacy of one treatment over others for a specific disease or condition. In such situations, investigators may utilize the network meta-analysis method by using studies with varying intervention groups to prioritize the effects of interventions. A recent study summarized the efficacy and acceptability of multiple antidepressant drugs for major depressive disorder (Cipriani et al., 2018). A meta-analysis can be used to assess the pattern, sufficiency, and stability of the effect size in the view of cumulative evidence. We demonstrated coronavirus disease (COVID-19) treatment uses in managing patients over time using a cumulative meta-analysis approach (Dubey et al., 2021a). In addition, a cumulative meta-analysis can also help to determine if the additional evidence is sufficient to establish the efficacy of an intervention or if further evidence does not change the effect size (Muellerleile & Mullen, 2006). The dose–response meta-analysis is useful for establishing the optimum cutpoint associated with the outcome. Multiple dose–response meta-analyses have been conducted to determine optimum body mass index levels or glycemic index associated with CVD outcomes (Dwivedi et al., 2020; Dwivedi et al., 2022). Furthermore, the meta-analysis approach can be utilized to determine the factors associated with heterogeneity in treatment responses (Abanto et al., 2024).

### Components of a meta-analysis

There are three main components, including the design phase, data collection phase, and analysis and reporting phase of a meta-analysis. These three components involve a total of 24 steps (Table 1). Table 2 provides biostatistical guidance for selecting evidence-based biostatistical methods for conducting meta-analysis, in accordance with the objective and steps involved in meta-analysis.

**Table 1:** Checklists for conducting and reporting an HQSR and meta-analysis.

Design phase	1	Develop a research question and conduct a preliminary search	PICO or POSE
	2	Sample size estimation and finalizing the research question	Use the sample size formula
	3	Select an evidence synthesis design	(a) umbrella review (b) scoping review (c) rapid review (d) HQSR (e) meta-analysis
	4	Define the objective of a systematic review and/or meta-analysis	(a) resolve conflicting findings (b) update the generalizability (c) varying interventions (d) pattern of effects (e) dose–response (f) prognostic evaluation
	5	Assemble a team	Include methodologists and topic experts
	6	Specify eligibility criteria	List inclusion and exclusion criteria
	7	Define and specify the search strategies	Ensure comprehensive (multiple search engines and references, contacts), systematic (step-by-step search implementation process), and reproducible (search paragraphs) search
	8	Develop a data collection sheet	Ensure completeness of data collection
	9	Specify methods for quality assessment	EBB selection of a tool
	10	Methods for data consistency and statistical analysis plan	EBB guided methods for (a) data conversion (b) data standardization (c) publication bias evaluation (d) evaluation of small size effects (e) heterogeneity measures (f) primary and secondary meta-analysis (g) heterogeneity assessment (h) validation analysis (i) data reporting and interpretations, and (j) list statistical software
	11	Develop the protocol and register it	PROSPERO website
Data collection phase	12	Conduct the search and finalize studies	Flowchart, double search validation
	13	Extract data and store it in the designed database	Double data entry if possible
	14	Evaluate the quality of each study	Apply the EBB tool for quality assessment
	15	Prepare the analytic dataset	Convert the master datasheet into a quantitative datasheet for analysis and data-sharing processes
Analysis and reporting phase	16	Conduct qualitative and descriptive analyses	Detailed descriptive analysis to understand potential factors for heterogeneity
	17	Evaluate publication and reporting biases	Funnel plot and small-sized effects
	18	Evaluate heterogeneity and choose whether to proceed with meta-analysis or not	Heterogeneity measures
	19	Apply the primary and secondary approaches for meta-analysis	(a) select CEM/FEM, fixed effects model, and REM (b) select REM methods (c) robust variance estimation, nonparametric or adjusted analysis
	20	Methods for heterogeneity assessment	LOOA, MR, Subgroup Analysis
	21	Sensitivity analysis	Validation analysis
	22	Finalize primary findings	Using collective methods (a) sample size and number of studies (b) high-quality studies (c) low heterogeneity (d) low publication bias
	23	Reporting effect size and intervals	Effect size with 95% confidence and predictive intervals
	24	Interpretation and transparent reporting	PRISMA and MOOSE

**Notes.**

PICO population, intervention, comparative group, Outcome POSE population, outcome, study design, exposure HQSR high-quality systematic review EBB evidence-based biostatistics PROSPERO prospective register of systematic reviews FEM fixed effect model CEM common effect model REM random effects model LOOA leave one out analysis MR meta-regression PRISMA preferred reporting items for systematic review and meta-analysis MOOSE meta-analysis of observational studies in epidemiology

**Table 2:** Biostatistical guidance for selecting standard and preferred methods for conducting a meta-analysis study.

**Issue**	**Standard method**	**EBB preferred method**
Conduct a meta-analysis or not	Conduct even with 2 or 3 studies	Compute sample size for meta-analysis or conduct meta-analysis with 10 or more studies
		
Meta-analysis for effect size estimation	Fixed effect or DL random effects models	HKSJ for a small number of studies; REML for a large number of studies
Network meta-analysis	REML	REML for continuous, GLM for rare outcomes or with high heterogeneity
Dose–response/correlation meta-analysis	Different methods	One-stage RCS model with non-fixed knots
Cumulative meta-analysis	Random effects model	Two-stage sample size weighted estimation of the random effects model, conduct with 20 or more studies
		
Standardized effect size for continuous outcome	Cohen’s d	Hedges’ g
Standardized effect size for a binary outcome	OR or RR	logOR for low or rare event rates or with heterogeneity; logRR with less heterogeneity
Heterogeneity assessment	*p*-value for the Cochrane Q test or I^2^ statistic<50% low heterogeneity	I^2^ statistic <25% (low heterogeneity ) and >50% (moderate or high heterogeneity)
Publication bias	Begg or Egger’s tests	Egger’s test for continuous outcome; Harbord’s test for binary outcome
Handling of publication bias	Restricted analysis	Cumulative meta-analysis and the Trim and Fill method
Sensitivity analysis for random effects	Different methods	Paule-Mandel method with Q-profile for 95%CI for continuous outcome /GLM for binary outcome
Heterogeneity assessment	Subgroup analysis	LOOA and meta-regression analysis
Multiple outcomes from the same studies	Individual outcome analysis	Multilevel meta-analysis

**Notes.**

EBB evidence-based biostatistics DL DerSimonian and Laird HKSJ Hartung-Knapp-Sidik-Jonkman REML restricted maximum likelihood RCS restricted cubic spline OR odds ratio RR risk ratio GLM generalized linear model LOOA Leave one-out analysis

#### Design phase

The investigators first need to define a research question. As per the research question, the investigators need to carry out a preliminary search and determine a rough estimate of eligible studies related to the topic for estimating the sample size (Valentine, Pigott & Rothstein, 2010). The sample size and power analysis for a meta-analysis study can be conducted using software such as PASS 2025 (NCSS, 2025) or R (Soerensen et al., 2023; Wetterslev et al., 2009; Hedges & Pigott, 2001), considering its importance in selecting meta-analysis *vs.* other systematic review designs for an evidence synthesis study. After that, the investigators need to refine and formulate a clear research question using PICO or POSE tools, or other tools, , conduct a sample size and power analysis (Jakobsen et al., 2014), develop search strategies with search engines, and specify data collection fields (Dwivedi & Shukla, 2020; Muka et al., 2020). At this phase, the investigators should develop a protocol that specifies the exposure/intervention, outcomes with their types, eligibility criteria based on study design, and factors affecting homogeneity (patient characteristics including type and stage of disease/condition, assessment of outcomes, type of intervention/exposure, missing data, follow-up, study characteristics including sampling methods including hospital-based, population-based or clinic-based, sample size, study location, study methods, quality, *etc.*), quality assessment methods, statistical analysis and reporting plan including methods for pooled summary estimation, assessment of heterogeneity, publication bias, sensitivity analysis, and interpretation and transparent reporting of study findings (Pigott & Polanin, 2020; Siddaway, Wood & Hedges, 2019).

#### Evidence search and data collection phase

At this phase, the investigators will execute the systematic review and abstract data from published studies as per the study protocol. The investigators need to demonstrate that the search is exhaustive and reproducible, and the data collection is comprehensive, with all measurements that can be used for conversion analysis and heterogeneity assessments. A study provides guidelines for conducting extensive and reproducible searches for conducting an HQSR (Muka et al., 2020; Siddaway, Wood & Hedges, 2019). In addition, the investigators need to grade each article using an appropriate tool specific to the included study design type (Dwivedi & Shukla, 2020). The data collection sheet should be in a format that can be shared upon publication with the journal.

#### Analysis and transparent reporting phase

In the analysis phase, the methodologists need to perform statistical procedures as appropriate with the meta-analysis objective. It may be worth preparing the meta-analysis codes that could be supplemented with the published article. The analysts need to demonstrate that data from all eligible studies are included in the pooled summary either through conversion or standardization methods. However, the conclusion should be driven by the analysis of high-quality, graded studies with limited heterogeneity and low publication bias, with consistent findings from sensitivity analyses (Haidich, 2010; Pigott & Polanin, 2020).

### Steps in data collection and data analysis

#### Building a team

A meta-analysis requires a team science approach in which at least 3–5 researchers, including a subject expert, a biostatistician with an interest in meta-analysis or a meta-analysis expert, and individuals for data extraction and quality assessments. The median number of authors included in a meta-analysis study was estimated to be 5 (Borah et al., 2017). A meta-analysis must include a subject expert and a meta-analysis expert. The subject expert assists in setting up the definition of exposure, outcome, search terms and their synonyms, eligibility criteria, and critical variables for data analysis, and data needed for exploring the heterogeneity while a methodologist assists in setting up the search phrase with Boolean operators, search restriction strategies, all sorts of quantitative data for conversion analysis and meta-analysis, methods for heterogeneity assessment, and appropriate methods for data analysis and adherence to reporting guidelines.

#### Evidence search

In a meta-analysis, one of the requirements is to obtain all possible research evidence related to the study objective. One of the major challenges to obtaining all articles in a literature search is that the search is not comprehensive and non-reproducible. Sometimes, even if the search is comprehensive, it may miss critical studies. In addition, investigators may end up with too many articles at the screening phase. Bramer et al. (2017) recommended that at least 3–4 search databases, such as Medline, Embase, Google Scholar, and Web of Science, be used to comprehensively produce all articles related to the topic, specifically if all search terms are specified correctly in the search strategies. Moreover, it has been identified that incorrect use of Boolean operators, inappropriate use of parentheses for combining search terms, syntax errors in search term truncation, missing important MeSH terms, typically not in free-texts, and missing synonyms of search terms are the main reasons for missing important articles from the search. To generate a reproducible search, it is recommended that at least two individuals conduct the search and compare their search strategies, search terms, and search statements to identify and resolve discrepancies to finalize search terms with appropriate statements to produce a reproducible search strategy (Siddaway, Wood & Hedges, 2019). Additionally, references of review studies on related research questions should also be screened for identifying any pertinent studies. The investigators performing the search should further learn using the “NOT” operator, restrict exclusion criteria appropriately, and advance search options to limit their screening articles.

#### Data extraction

Quantitative data extraction is required for meta-analysis. In most clinical meta-analysis studies, some data including age, gender, ethnicity, disease type, comorbidities, disease duration, study location, year of publication, study sample criteria, study size, sub-study design, measurements for outcomes, duration of intervention and dose, exposure and outcome definitions, completed or early truncated studies, methods/technology used for outcome assessments, and unadjusted and adjusted estimates are often required (Pigott & Polanin, 2020; Siddaway, Wood & Hedges, 2019). The investigators should prepare a database to record all these essential data variables, depending on their research questions. In addition, other appropriate data should be extracted from a study. To avoid any inconsistencies in data recording, it is better to involve two investigators to independently extract data to resolve any conflicts in data entries and abstraction. During the data extraction, the major challenges are inconsistent reporting of statistics for the same variables. For example, studies might report means with standard errors, medians with interquartile ranges, mean differences with 95% confidence intervals, mixed data entries (continuous *vs.* categorized), and effect sizes in relative measures or absolute measures. In such situations, investigators should extract an appropriate set of data to conduct conversion analysis so that the most critical data can be used in the statistical analysis (Abbas, Hefnawy & Negida, 2024). Sometimes, the data may not be provided in the table or texts but rather reported in the graphs or images. In this case, the investigators may use web-based tools to extract data from graphs or images. If studies report multiple follow-up datasets, then the investigators should extract all follow-up datasets for statistical analyses (Pigott & Polanin, 2020). The investigators should attempt to minimize missing data, either due to language or non-availability of full texts, or non-reporting, by using a translator or contacting the authors of the published articles (Muka et al., 2020).

#### Grading of eligible studies

Since HQSR and meta-analysis depend on the evidence synthesis based on quality studies, quality assessment is a necessary step in conducting a meta-analysis. There are multiple tools available for evaluating the quality of included studies in a systematic review, depending on the study design and objective of the study. Investigators must pick the right tool for quality assessment. Evidence-based biostatistics approach should be used to decide the preferred methods of grading eligible studies (Dwivedi, Ahmed & Dubey, 2025a; Dwivedi & Shukla, 2020). Although most meta-analysis studies report the quality of included studies using a methodological assessment tool, investigators typically make conclusions based on all studies, not on high-quality studies. It is recommended that the influence of high-quality studies on conclusions should be reported through sensitivity analyses.

#### Data format for primary analysis

The first step is to reduce missing data on primary outcomes and exposures using conversion methods and standardize data across studies. One of the major challenges in data analysis is that data reporting from different studies is not in a uniform format. For example, different assays, methods, instruments, or technologies may have been used for data collection in different studies. In such situations, the analysts should standardize the data using Cohen’s or other methods for meta-analysis (Abbas, Hefnawy & Negida, 2024). Similarly, relative ratio measures (odds ratio, risk ratio, hazard ratio) and mean differences are reported in different studies. It is required to convert all data to the same effect size measurement for pooled analyses (Higgins, Li & Deeks, 2023).

#### Descriptive analysis

Using descriptive analysis, the investigators may want to assess distribution, zero or limited events in any studies, variation in the sizes of the studies and designs, consistencies in the conclusions, and heterogeneity measurements in the effect size across studies. Three common statistics, known as the Q-test, H-statistic, and I^2^ statistic, are used to assess heterogeneity in the effect sizes across studies. Q-test informs us whether the heterogeneity between studies is significantly different than zero or not, while H-statistic informs us about the overall variability compared to variation within the studies by generating a ratio between estimated total variation (between and within studies) using the random effects model compared to the variation within studies using the fixed effect model. The I^2^ statistic quantifies the degree of inconsistencies across studies and compares the Q-value to its expected value assuming homogeneity across studies (Higgins et al., 2003). The I^2^ statistic can be used to compare the heterogeneity in different meta-analysis studies and can be supplemented by an uncertainty interval. Its interpretation is intuitive and easy, irrespective of the type of outcome and choice of effect size measure. I^2^ < 25% indicates a low presence of heterogeneity, between 25%–75% (moderate), and ≥75% (high presence of heterogeneity) (Ioannidis, Patsopoulos & Evangelou, 2007).

#### Evaluate publication and reporting bias

Negative or non-significant studies are likely to be unpublished, creating publication bias issues in data synthesis. Using funnel plots, the methodologists can identify if there are missing studies that may influence the overall estimate of the outcome. In the presence of publication bias, the methodologists need to use the nonparametric trim and fill method to provide a bias-corrected effect size (Shi & Lin, 2019). In addition, the asymmetric funnel plot can also help in identifying missing studies, especially small-sized studies. Appropriate statistical tests can also be used to detect whether the results of smaller studies differ systematically from the results of larger studies. Egger’s test is preferred for meta-analysis with continuous outcomes, while Harbord’s test is preferred for meta-analysis with binary outcomes (Schwab, Kreiliger & Held, 2021). In the case of small-study effects, it is recommended to conduct a meta-analysis for large sample-size studies using the cutoff for large-sized studies determined with a cumulative meta-analysis approach (Muellerleile & Mullen, 2006) and conduct sensitivity analyses (IntHout et al., 2015).

#### Selecting the appropriate statistical approach for meta-analysis

There are three approaches: (a) fixed effect or common effect model (CEM/FEM), (b) fixed effects model, and (c) random effects model (REM) (Borenstein et al., 2010). Most often, researchers either use the CEM or REM depending on the presence of quantitative heterogeneity (either measured by the I^2^ statistic or the Q-statistic) or qualitative heterogeneity (in terms of methodological inconsistencies). The CEM/FEM assumes that all the effect sizes from different studies represent a common true effect, and a weighted procedure can be used to estimate the pooled or average effect of a fixed true effect size. The effect sizes vary across studies due to sampling errors. In contrast, a fixed effects model assumes that all the effect sizes from different studies are a random selection from a known population representing an unknown true effect. The effect sizes vary across studies due to random errors. In the fixed effect or fixed effects model, the overall variation in the pooled effect size is explained through variation within each study. Although the fixed effect and fixed effects models are often interchanged due to the same results, the interpretations are different between these models. Moreover, fixed effects models allow us to conduct meta-regression analysis compared to the FEM/CEM model. The REM assumes that all effect sizes represent a random sample of effect sizes from all possible studies and that variation in the effect sizes is due to study characteristics. In the REM, the overall variation in the pooled effect size is explained through variations within each study and between studies. The FEM/CEM gives higher weight to the studies with smaller variances or studies with larger sample sizes, whereas the REM gives relatively similar weights across studies. Since meta-analysis estimates should be based on high-quality studies, which are typically represented by larger studies, FEM has been preferred by some methodologists. In contrast, small-sized studies may also be included in evidence synthesis, and therefore, all studies should be given relative weights, and the REM should be preferred. It is hard to assume that there is no heterogeneity between studies, and therefore, it is recommended to use REM except for the low number of studies. In fact, the REM severely underestimates the confidence range where the true effect lies. Therefore, it is recommended to estimate and report predictive intervals for the estimates obtained using the REM (Borenstein et al., 2010; Dettori, Norvell & Chapman, 2022). The predictive interval provides a confidence range where the true effect will lie outside of the studies (IntHout et al., 2016). Unfortunately, limited studies report predictive intervals for the estimates owing to non-significant results.

#### Selecting appropriate methods in REM

There are several methods available for obtaining pooled estimates in REM analysis. Evidence-based biostatistics approach should be used for deciding the preferred methods of REM (Table 2) (Dwivedi & Shukla, 2020; Veroniki et al., 2016).

#### Methods for heterogeneity assessments

Regardless of the use of REM or FEM, the conclusions of meta-analysis must be done in the absence of significant heterogeneity (I^2^ < 50%) and publication bias. Statistical methods such as leave-one-out analysis (LOOA), meta-regression analysis (MR), and subgroup analysis are typically used to identify the cause of heterogeneity between studies. In LOOA, one or two studies at a time are excluded from the pooled analysis to determine any outlier study that can minimize the heterogeneity (Patsopoulos, Evangelou & Ioannidis, 2008). The methodologists need to summarize reasons why the exclusion of one or two studies reduced the heterogeneity completely. Sometimes, excluding a study does not minimize the heterogeneity completely. In that case, the methodologists can perform a meta-regression analysis based on the extracted patient characteristics (comorbidities, age, sex, duration of treatments, dose, *etc.*) from original studies. The subgroup analyses based on study characteristics (study design, sample size, study location, study methods, *etc.*) may provide a stratification factor that can minimize the heterogeneity (Glisic et al., 2023). In our recent meta-analysis for determining the relationship between cerebrospinal fluid Aβ42 and cognitive changes, we identified that the exclusion of a study could eliminate the heterogeneity in the effect size using an LOOA (Abanto et al., 2024). Similarly, in another meta-analysis for estimating the prevalence of adverse pregnancy outcomes in COVID-19 patients, stratifying the prevalence estimates by geographic location, considering the global pandemic resolved heterogeneity issues (Dubey et al., 2020).

#### Sensitivity analysis

Sensitivity analyses are required to validate the reproducibility of the findings (Dwivedi, 2022). Typically, sensitivity analyses are used to evaluate the consistencies and robustness of the findings obtained through primary data analysis by demonstrating the influence of the study specific variables (methodological or non-methodological), assumptions, or inclusion criteria on effect estimates or heterogeneity. Sensitivity analysis related to methodological variables may be based on restricting analysis to studies without low-quality studies, methods conversion, different methods for outcome and exposure assessments, and unadjusted estimates, publication bias, inclusion of studies with false findings or biased effect sizes, *etc.* A meta-analysis including early premature studies, early truncated trials, unadjusted effect sizes from observational studies, and false findings may produce biased pooled effect sizes and conclusions (Bassler et al., 2010; Schuit et al., 2015). Meta-analysis evaluating HRT and risk of CVD reported a significantly reduced risk of CVD associated with HRT in postmenopausal women (Grodstein et al., 2000). However, restricting original studies that adjusted the socioeconomic status in their analyses produced no association between hormonal use and reduced risk of CVD in postmenopausal women (Humphrey, Chan & Sox, 2002), indicating inclusion of studies with false or biased findings may significantly affect the conclusions of a meta-analysis study. In a meta-analysis evaluating the relationship between maternal PCOS and neuropsychiatric outcomes in offspring, we validated findings by conducting sensitivity analyses on restricted studies based on PCOS and autism spectrum disorder diagnoses and conversion effect sizes (Dubey et al., 2021b).

#### Interpretation and transparent reporting

PRISMA and MOOSE checklists specify the step-by-step methods for transparent reporting of meta-analysis studies (Brooke, Schwartz & Pawlik, 2021; Muka et al., 2020; Page et al., 2021). The interpretations of the findings should be provided in the context of consistency in the findings, considering the sample size, with a good number of high-quality studies with low or negligible heterogeneity and low publication bias. An unexplained large heterogeneity provides no meaningful interpretation. It is strongly recommended to report the effect size along with 95% confidence and predictive intervals (IntHout et al., 2016).

### Writing steps of a meta-analysis publication

The introduction section of a meta-analysis must specify the study objective, including (a) resolving conflicting findings, (b) increasing generalizability, (c) intervention effects with varying groups, (d) pattern of effects, sufficiency, and stability, (e) dose–response relationship, and (f) identifying prognostic factors. The methods section should specify steps considered for the comprehensiveness of data search, reproducibility of data search, sample size and power analysis, eligibility criteria, protocol registration, outcomes and exposures, authenticity and completeness of data collection for primary and heterogeneity exploration analyses, grading evaluation of included studies, and statistical analysis. The statistical analysis section should describe statistical methods and procedures used for (a) standardization methods for determining uniformity in data outcomes and exposures, (b) conversion analysis for completeness of data, (c) descriptive analysis, (d) methods for evaluating publication bias including small size effects, (e) heterogeneity estimation, (f) estimating pooled effects, (g) exploration of heterogeneity analysis, (h) sensitivity analysis, and (i) interpretation and reporting of findings including effect size, confidence interval, and predictive interval. In addition, statistical software and the false positive level to be considered statistically significant results need to be specified in the statistical analysis section. The results section should include a description of the flowchart for study selection, qualitative/descriptive data analysis, findings from primary and sensitivity/validation analyses, and additional subgroup analyses. The discussion section typically includes (a) a summary of the key findings in the first paragraph, (b) reasons for heterogeneity in the estimated effect size with reduced or eliminated heterogeneity in the second paragraph, (c) the level of generalizability using subgroup analysis in the second paragraph, (d) the quality of evidence produced based on sensitivity and validation analyses in the third paragraph, (e) potential mechanisms and implications of observed findings in the fourth paragraph, and (f) limitations, strengths, and future expansion of study findings in the fifth paragraph. The checklists for conducting an HQSR and meta-analysis are included in Table 1.

## Conclusions

There are various types of review and evidence synthesis studies in the literature. Since inappropriate selection and conduct of an evidence synthesis method adversely affect the credibility of the scientific findings and informed decision processes for future research and clinical decisions, this guidance document is prepared for investigators to select an appropriate type of evidence synthesis method and related steps to follow. Accordingly, the selection of an appropriate type of evidence synthesis study depends on the study objective, hypothesis, sample size, number of original studies, heterogeneity in studies, data reporting across studies, and time and resources. Since it is not always possible to convert any systematic review to an HQSR, the investigators may prefer to conduct a high-quality scoping review with or without a meta-analysis. Suppose investigators do not adhere to systematic review guidelines and related methodological rigor, they may refer to their study as a general evidence synthesis study rather than a systematic review. This report also clarifies the different objectives of a meta-analysis and the importance of proper labeling of a systematic review design. In addition, this guidance document also facilitates the steps to write various sections of a systematic review study, including a statistical analysis section for meta-analysis. Overall, this document may help resolve heterogeneity in practice for the conduct of a systematic review and may promote evidence-based and value-based biostatistics practice in evidence synthesis (Dwivedi, Ahmed & Dubey, 2025a; Dwivedi et al., 2025b). Although an updated systematic review is required in medical research as the evidence accumulates over time, it is required that researchers receive proper education and training on the conduct and reporting of appropriate systematic reviews. Following the reported evidence-based biostatistics practice checklists for conducting and reporting a meta-analysis study is necessary for producing high-quality evidence for research and clinical practices.

## Abbreviations

HQSR: high-quality systematic review
PRISMA: preferred reporting items for systematic review and meta-analysis
MOOSE: meta-analysis of observational studies in epidemiology
RCT: randomized controlled trial
IPD-MA: individual participant data meta-analysis
AD-MA: aggregated participant data meta-analysis
PICO: population, intervention, comparative group, outcome
POSE: population, outcome, study design, Exposure
EBB: evidence-based biostatistics
PROSPERO: prospective register of systematic reviews
FEM: fixed effect model
CEM: common effect model
REM: random effects model
LOOA: leave one out analysis; MR: meta-regression

## Additional Information and Declarations

### Data Availability

The following information was supplied regarding data availability: This is a Methods article.
